# RNAstrand: reading direction of structured RNAs in multiple sequence alignments

**DOI:** 10.1186/1748-7188-2-6

**Published:** 2007-05-31

**Authors:** Kristin Reiche, Peter F Stadler

**Affiliations:** 1 Bioinformatics Group, Dept. of Computer Science, and Interdisciplinary Center for Bioinformatics, University of Leipzig, Härtelstraße 16-18, D-04107 Leipzig, Germany; 2 Institute for Theoretical Chemistry, University of Vienna, Währingerstraße 17, A-1090 Wien, Austria; 3 Santa Fe Institute, 1399 Hyde Park Rd., Santa Fe, NM 87501, USA

## Abstract

**Motivation:**

Genome-wide screens for structured ncRNA genes in mammals, urochordates, and nematodes have predicted thousands of putative ncRNA genes and other structured RNA motifs. A prerequisite for their functional annotation is to determine the reading direction with high precision.

**Results:**

While folding energies of an RNA and its reverse complement are similar, the differences are sufficient at least in conjunction with substitution patterns to discriminate between structured RNAs and their complements. We present here a support vector machine that reliably classifies the reading direction of a structured RNA from a multiple sequence alignment and provides a considerable improvement in classification accuracy over previous approaches.

**Software:**

RNAstrand is freely available as a stand-alone tool from  and is also included in the latest release of RNAz, a part of the Vienna RNA Package.

## Introduction

Genome wide computational screens for structured ncRNA genes in mammals [1-3], urochordates [4], nematodes [5], and drosophilids [6] resulted in tens of thousands putative structured ncRNAs. Functional and structural annotation of these predictions thus becomes a pressing problem. Evidence for evolutionary conservation of RNA structure alone usually does not distinguish very well between the two possible reading directions. This information, however, is crucial already for the most basic annotation information. Direction information is needed e.g. to determine whether a conserved RNA motif is intronic, located within a coding sequence or an untranslated exon, an independent ncRNA, or one of the many classes of small RNAs associated with other transcripts [7].

The RNAstrand tool is designed specifically to predict the reading direction of a multiple sequence alignment *under the assumption *that the alignment contains an evolutionary conserved RNA secondary structure. Our task at hand is a conceptually simple two class prediction problem for which we employ a support vector machine (SVM) [8]. The basic idea is to devise descriptors that utilize both the small asymmetry in the energy rules [9] and the asymmetric effect of GU base pairs.

## 1 Methods

### 1.1 Selection of descriptors

Small differences in the measured folding energies between an RNA molecule and its reverse complement are captured by corresponding small asymmetries in the standard energy model used by thermodynamic folding algorithms [9,10]. These differences distinguish the two reading directions even in the absence of GU pairs. In addition, GU pairs have an asymmetric effect in multiple sequence alignments: Suppose a particular pair of alignment columns exhibits a GC → GU substitution in one reading direction; this preserves base pairing and hence is consistent with a conserved structure. The reverse complement of the same alignment, however, displays a GC → AC substitution which is inconsistent with a conserved base pair. The patterns of structure conservation, and hence the consensus structure and its associated average folding energy, as computed by the RNAalifold algorithm [11], thus differ between the reading directions. In contrast, compensatory mutations, such as GC → AU do not provide strand-specific information.

The effects of both the asymmetries of the energy rules and of the GU base pairs are conveniently captured in terms of thermodynamic quantities, more precisely, in terms of the folding energies of the consensus structure and the individual folding energies of a set of aligned RNAs. These parameters can be computed much more reliably than quantities that have to be derived from predicted base pairs due to the limited accuracy of the structure prediction algorithms on individual sequences [12]. We avoid the use of sequence motifs (e.g. [13]), since this bears the danger that the SVM is biased to the ncRNA families in the training set and fails to distinguish plus and minus strands of other structured ncRNAs.

Here we use:

1) Average of the folding energies of the individual sequences contained in the alignment, computed by the minimum energy folding program RNAfold of the Vienna RNA Package, version 1.6 [14] (***meanmfe***).

2) Mean of the energy *z*-scores of the individual sequences contained in the alignment (***meanz***). The *z*-score is defined as *z *= (*E *- E¯
 MathType@MTEF@5@5@+=feaafiart1ev1aaatCvAUfKttLearuWrP9MDH5MBPbIqV92AaeXatLxBI9gBaebbnrfifHhDYfgasaacH8akY=wiFfYdH8Gipec8Eeeu0xXdbba9frFj0=OqFfea0dXdd9vqai=hGuQ8kuc9pgc9s8qqaq=dirpe0xb9q8qiLsFr0=vr0=vr0dc8meaabaqaciaacaGaaeqabaqabeGadaaakeaacuWGfbqrgaqeaaaa@2DD7@)/*σ*, where E¯
 MathType@MTEF@5@5@+=feaafiart1ev1aaatCvAUfKttLearuWrP9MDH5MBPbIqV92AaeXatLxBI9gBaebbnrfifHhDYfgasaacH8akY=wiFfYdH8Gipec8Eeeu0xXdbba9frFj0=OqFfea0dXdd9vqai=hGuQ8kuc9pgc9s8qqaq=dirpe0xb9q8qiLsFr0=vr0=vr0dc8meaabaqaciaacaGaaeqabaqabeGadaaakeaacuWGfbqrgaqeaaaa@2DD7@ and *σ *are mean and standard deviation of the folding energy distribution of shuffled (permuted) sequences. We use here the same SVM-regression procedure as RNAz [15] to estimate the *z*-scores from the sequence composition to avoid the time consuming sampling of shuffled alignments.

3) Folding energy of the consensus secondary structure of the alignment computed by RNAalifold (***consmfe***). The parameter is defined as the optimal average of the folding energies that can be achieved when all aligned sequences simultaneously fold into the same structure.

4) Structure conservation index (***sci***), which is defined as the ratio of the consensus folding energy and the average of the folding energies of the individual sequences, i.e. *sci *= *consmfe/meanmfe*, [15]. An *sci *close to 1 indicates perfect structure conservation, while alignments without structural conservation yield values close to 0. A more detailed discussion of the *sci *can be found in [16] in the context of RNA alignment.

The first two descriptors assess the thermodynamic stability of the folds, while the last two evaluate structural conservation.

The reading direction of a structured ncRNA can be identified by evaluating the *differences *of the above descriptors between both strands. To be precise, the difference Δ*x *of descriptor *x *is defined as Δ*x *= *x*_+ _- *x*_-_, where *x*_+ _denotes the value of *x *in reading direction of the input alignment and *x*_- _the value of *x *in the reverse complementary alignment. Hence, Δ*meanmfe *and Δ*meanz *capture the energetic differences between both strands, while Δ*consmfe *and Δ*sci *describe the differences in structure conservation.

**Figure 1:**
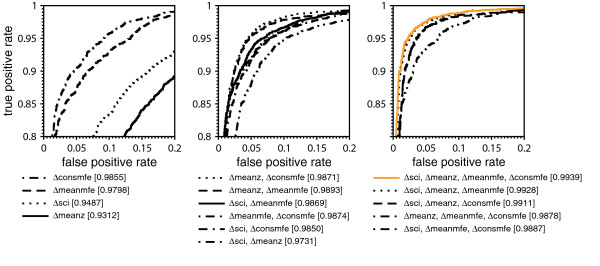
**Receiver operating characteristic of all descriptor combinations**. Receiver operating characteristic (ROC) for all descriptor combinations. Corresponding *AUC *is given in brackets. ROC curves were computed by a 5-fold cross-validation on the training data set using plotroc.py of the libsvm 2.8 package [18] after an optimal SVM parameter set was chosen by grid.py. True positive and false positive rates are calculated by interpreting the SVM decision values. Prediction accuracies as plotted here are larger compared to accuracies in Table 1 as even though cross-validation ensures that training and testing is done on different alignments some sequences may occur in the training as well as in the test alignments. In contrast, accuracies in Table 1 are based on test alignments which do not contain any sequence attending at a training alignment.

The proportion of true positive and false positive rate (ROC curve) for each combination of descriptors is summarized in Fig. 1. It reveals which combination of descriptors achieves optimal classification of the alignments. The ROC curves can be evaluated by the area under the curve (*AUC*), which states the similarity of the ROC curve to a step function. The steeper the true positive rate increases while staying at its maximum value for different values of false positive rates, the better the input alignments can be separated. The best *AUC *of 99% is achieved when all four descriptors are taken.

Note, that although *sci *= *consmfe*/*meanmfe*, i.e., these three quantities are not independent, this is not the case for their differences. Δ*sci *cannot be computed from Δ*consmfe *and Δ*meanmfe*. Furthermore, for alignments where the structural conservation is very high in both reading directions the strand of the ncRNA cannot be inferred by Δ*sci *alone. But the difference of consensus structure stability, which is measured by Δ*consmfe *may still predict the strand correctly.

Same holds for Δ*meanz *and Δ*meanmfe*. Both measure the folding energy differences of the individual sequences, but do not capture identical features of the input alignment nor can be transformed into each other. The mean *z*-score compares the average stability of individual sequences to a random control set. Whereas the mean of minimum free energies of individual sequences specifies the actual observed minimum free energies. The difference in *z*-scores describes the relative loss of stability compared to a random control set. It quantifies that the input alignment swaps from very stable to unstable between both strands. The difference in minimum free energy, on the other hand, is able to specify small changes in energies, which is needed to find the correct reading direction of the ncRNA in case both reading directions result in very stable structures. An example are miRNAs, which are very stable on both strands but are nevertheless successfully classified by RNAstrand. Hence, all four descriptors carry different information. 

The significance of differences in folding energies depends on the number of sequences in the input alignment, denoted by *n*, and on sequence variation. The latter is conveniently quantified as the average pairwise sequence identity *H *of both reading directions.

The strongest strand information comes from GU base pairs which are unpaired in the reverse complementary alignment. Hence, the relevance of differences depends also on the overall number of GU base pairs in the consensus structure. Therefore, we introduce

**Figure 2:**
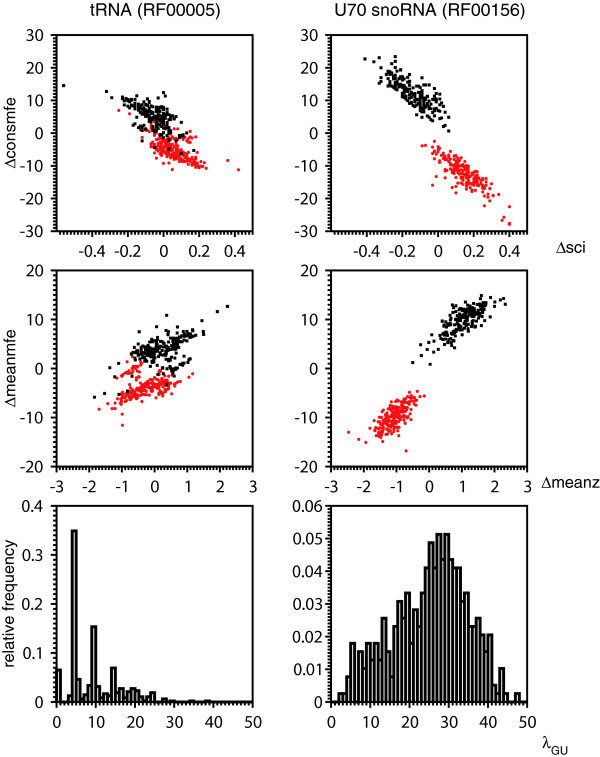
**GU base pair dependency**. Scatter plots depicting separability between both strands depending on GU base pair content (histograms). Red data points denote alignments in the reading direction of the ncRNA, while black data points belong to their realigned reverse complements. Alignments of tRNAs and U70 snoRNAs do not have significantly different number of sequences nor differ significantly in mean pairwise identity (see Additional file 1). That alignments in reading direction of U70 snoRNA are well separated from their reverse complements compared to alignments containing tRNAs is due to high content of GU base pairs in the secondary structure of U70 snoRNAs.

λGU=(nGU+nall++nGU−nall−)×100,
 MathType@MTEF@5@5@+=feaafiart1ev1aaatCvAUfKttLearuWrP9MDH5MBPbIqV92AaeXatLxBI9gBaebbnrfifHhDYfgasaacH8akY=wiFfYdH8Gipec8Eeeu0xXdbba9frFj0=OqFfea0dXdd9vqai=hGuQ8kuc9pgc9s8qqaq=dirpe0xb9q8qiLsFr0=vr0=vr0dc8meaabaqaciaacaGaaeqabaqabeGadaaakeaaiiGacqWF7oaBdaWgaaWcbaGaem4raCKaemyvaufabeaakiabg2da9iabcIcaOmaalaaabaGaemOBa42aa0baaSqaaiabdEeahjabdwfavbqaaiabgUcaRaaaaOqaaiabd6gaUnaaDaaaleaacqWGHbqycqWGSbaBcqWGSbaBaeaacqGHRaWkaaaaaOGaey4kaSYaaSaaaeaacqWGUbGBdaqhaaWcbaGaem4raCKaemyvaufabaGaeyOeI0caaaGcbaGaemOBa42aa0baaSqaaiabdggaHjabdYgaSjabdYgaSbqaaiabgkHiTaaaaaGccqGGPaqkcqGHxdaTcqaIXaqmcqaIWaamcqaIWaamcqGGSaalaaa@5120@

as last descriptor. nGU+(nGU−)
 MathType@MTEF@5@5@+=feaafiart1ev1aaatCvAUfKttLearuWrP9MDH5MBPbIqV92AaeXatLxBI9gBaebbnrfifHhDYfgasaacH8akY=wiFfYdH8Gipec8Eeeu0xXdbba9frFj0=OqFfea0dXdd9vqai=hGuQ8kuc9pgc9s8qqaq=dirpe0xb9q8qiLsFr0=vr0=vr0dc8meaabaqaciaacaGaaeqabaqabeGadaaakeaacqWGUbGBdaqhaaWcbaGaem4raCKaemyvaufabaGaey4kaScaaOGaeiikaGIaemOBa42aa0baaSqaaiabdEeahjabdwfavbqaaiabgkHiTaaakiabcMcaPaaa@37F9@ denotes the number of GU base pairs in the consensus secondary structure of the reading direction of the input alignment (reverse complement of the input alignment), and nall+
 MathType@MTEF@5@5@+=feaafiart1ev1aaatCvAUfKttLearuWrP9MDH5MBPbIqV92AaeXatLxBI9gBaebbnrfifHhDYfgasaacH8akY=wiFfYdH8Gipec8Eeeu0xXdbba9frFj0=OqFfea0dXdd9vqai=hGuQ8kuc9pgc9s8qqaq=dirpe0xb9q8qiLsFr0=vr0=vr0dc8meaabaqaciaacaGaaeqabaqabeGadaaakeaacqWGUbGBdaqhaaWcbaGaemyyaeMaemiBaWMaemiBaWgabaGaey4kaScaaaaa@332D@ and nall−
 MathType@MTEF@5@5@+=feaafiart1ev1aaatCvAUfKttLearuWrP9MDH5MBPbIqV92AaeXatLxBI9gBaebbnrfifHhDYfgasaacH8akY=wiFfYdH8Gipec8Eeeu0xXdbba9frFj0=OqFfea0dXdd9vqai=hGuQ8kuc9pgc9s8qqaq=dirpe0xb9q8qiLsFr0=vr0=vr0dc8meaabaqaciaacaGaaeqabaqabeGadaaakeaacqWGUbGBdaqhaaWcbaGaemyyaeMaemiBaWMaemiBaWgabaGaeyOeI0caaaaa@3338@ are the numbers of all base pairs in the consensus structure of the corresponding reading direction. Fig. 2 shows that alignments in the reading direction of a tRNA can not as easy be separated from the reverse complementary alignments by evaluating only Δ*meanmfe*, Δ*meanz*, Δ*consmfe *and Δ*sci *as it is the case for alignments containing U70 snoRNAs. The majority of tRNAs have around 0–5% GU base pairs in their consensus secondary structure. (The percentage of GU pairs is roughly *λ*_*GU*_/2.) In contrast, the majority of U70 snoRNAs have 10% to 20% GU base pairs in their consensus structure. *λ*_*GU *_allows the SVM to find suitable classification values depending on the fraction of GU base pairs. Therefore, U70 snoRNAs as well as tRNAs are classified correctly with high accuracies (U70: 1.0, tRNA: 0.94).

We regard GU base pair fraction rather of the consensus structure than of the predicted structures of the single sequences, as the structure prediction of RNAalifold is based on evolutionary information of a set of sequences and hence produces a fold more similar to the real structure than RNAfold is able to predict from one single sequence. We did not introduce the difference of GU base pairs as a descriptor, because the error rate of such an descriptor depends largely on the correctness of the predicted secondary structure. Small errors in structure prediction have a large impact on the difference of GU base pairs. In contrast, the difference in structure stability and conservation regards all base pairs and hence depends only very weakly on the correctness of individual base pairs.

In summary, the SVM classification is based on seven descriptors, of which four, Δ*meanmfe*, Δ*meanz*, Δ*consmfe *and Δ*sci *directly measure differences between the reading directions, while the remaining three, *n*, *H*, and *λ*_*GU *_provide information on the structure of the input alignment that allow the SVM to interpret the significance of strand differences.

### 1.2 Training of Support Vector Machine

Alignments for training were taken from the same sources as in [15] including representatives for rRNAs, spliceosomal RNAs, tRNAs, miRNAs, small nucleolar RNAs, nuclear RNaseP and SRP RNA. Sequence similarity in this data set ranges from 47% to 99% mean pairwise identity in alignments of 40 nt to 400 nt length and of 2 to 6 sequences. The detailed distributions of mean pairwise identity, length, number of sequences and GU base pair content are given in the supplementary material (see Additional file 1). A total of 5886 ClustalW alignments, approximately equally representing these ncRNA families, were used for training after removing alignments that were not recognized as structured RNA by RNAz in both reading directions. This data set was splitted into two subsets of equal size, namely the positive and negative training set. Alignments in the negative training set were transformed to the reverse complement and realigned with ClustalW as opposed to take just the reverse complementary alignment of the structured RNA.

The number of sequences a training alignment contains is limited to 6 as the SVM regression procedure to estimate the *z*-scores is trained with alignments of maximal 6 sequences [15]. In case an alignment has more than 6 sequences a subalignment with optimal mean pairwise identity may be chosen with the perl script rnazWindow.pl [17] of the RNAz package.

We use libsvm 2.8 [18] with SVM type C_SVC, a radial basis function (RBF) kernel, probability estimates and descriptor vectors scaled linearly to the interval [-1, +1]. The scaling avoids that descriptors which have a large variance dominate the classification. The values for the RBF kernel parameters *γ *and *C *were identified by a grid search in the parameter space applying grid.py of the libsvm 2.8 package with a 5-fold cross-validation on the training data. Maximal prediction accuracy is achieved with parameters *C *= 128 and *γ *= 0.5.

The SVM returns an estimated class probability *p*, that the ncRNA is found in the reading direction of the input alignment. We convert *p *into a score *D *= 2*p *- 1, so that *D *≈ +1 means "RNA in reading direction of input alignment" while *D *≈ -1 means "RNA is reverse complement of input alignment".

## 2 Results

### 2.1 Testing the classifier

Classification performance is evaluated using 30920 automatically generated ClustalW alignments of 313 of the 503 ncRNA families from RFAM (version 7.0). All sequences attending at the training alignments were excluded from the test set. For each family at most 500 ClustalW alignments were randomly constructed each for 2 to 6 sequences, resulting in maximal 2500 alignments for a family. Since the alignments which were taken to train the SVM are no longer than 400 nt, have a minimal pairwise sequence identity of 60% and contain maximal six sequences, test alignments were created which meet the same criteria. For alignments which do not fall into those ranges probability estimates of the SVM need to be regarded with certainty. 8 families had no alignments between 40 and 400 nt and were hence discarded from the test set. 67 families are not included because they consist of only one or two sequences. 2 families had no sampled alignments with a mean pairwise sequence identity larger than 60%. Lastly, the sampled alignments of 113 families were not recognized as ncRNA by RNAz on at least one reading direction and were also discarded from the test data set. A list of families excluded from the test data can be found in the supplementary material (see Additional file 1). All alignments in the test set were used as positive test cases and their realigned reverse complements as negative test cases.

Table 1 lists the classification rates for different threshold values *c*, i.e., classifying the RNA as "plus strand" for *D *> *c *and as "minus strand" for *D *< -*c*, while -*c *≤ *D *≤ *c *is interpreted as "undecided". We observe only a negligible loss of accuracy when *c *is increased from 0 to 0.9. The distribution of *D *(see Additional file 1) demonstrates that the majority of alignments are classified correctly with high probability. However, RNAstrand fails to predict the correct reading direction of 53 families (e.g. 7SK). The predicted secondary structure of the reverse complementary alignment is much more stable for these examples than the ncRNA itself (see Additional file 1). On the other hand, RNAstrand is able to reliably capture the reading direction of most ncRNAs for which no representative was given in the training set, including RNase MRP, IRES, SECIS and 5.8S rRNA, which makes it suitable to predict the reading direction of novel ncRNA families.

**Table 1:** Evaluation of RNAstrand.

				*c *= 0			*c *= 0.5			*c *= 0.9		
ncRNA type	*N*_*a*_	*N*_*c*_	*A*	*A*_+_	*A*_-_	*A*	1-*A*-*u*	*u*	*A*	1-*A*-*u*	*u*	*A*(RNAz)

Alignments classified as structured RNA by RNAz												

5S rRNA	413	1	**0.990**	0.993	0.988	0.978	0.006	0.016	0.958	0.000	0.042	**0.973**
5.8S rRNA	146	1	**0.932**	0.932	0.932	0.894	0.055	0.051	0.733	0.024	0.243	**0.904**
tRNA	286	1	**0.948**	0.948	0.948	0.886	0.017	0.096	0.621	0.009	0.371	**0.535**
miRNA	1875	43	**0.981 **[0.241]	0.979 [0.246]	0.982 [0.238]	0.965 [0.261]	0.009 [0.171]	0.026 [0.147]	0.906 [0.373]	0.001 [0.003]	0.094 [0.372]	**0.187 **[0.376]
snoRNA (C/D)	946	71	**0.780 **[0.376]	0.785 [0.374]	0.775 [0.389]	0.732 [0.411]	0.190 [0.363]	0.078 [0.256]	0.618 [0.431]	0.147 [0.286]	0.235 [0.416]	**0.654 **[0.446]
snoRNA (H/ACA)	3066	53	**0.909 **[0.198]	0.908 [0.198]	0.909 [0.199]	0.882 [0.255]	0.062 [0.160]	0.056 [0.184]	0.823 [0.352]	0.021 [0.039]	0.156 [0.339]	**0.899 **[0.283]
spliceos. RNA	896	6	**0.877 **[0.252]	0.885 [0.251]	0.868 [0.254]	0.831 [0.327]	0.086 [0.212]	0.083 [0.118]	0.735 [0.322]	0.042 [0.125]	0.222 [0.202]	**0.835 **[0.257]
euk. SRP RNA	891	1	**0.997**	0.998	0.996	0.992	0.001	0.007	0.972	0.000	0.028	**0.841**
nucl. RNaseP	31	1	**0.694**	0.710	0.677	0.613	0.274	0.113	0.387	0.081	0.532	**0.290**
RNase MRP	140	1	**0.989**	0.986	0.993	0.982	0.000	0.018	0.961	0.000	0.039	**0.500**
IRES	170	8	**0.715 [0.453]**	0.718 [0.455]	0.712 [0.452]	0.647 [0.469]	0.200 [0.424]	0.153 [0.339]	0.597 [0.448]	0.106 [0.433]	0.297 [0.402]	**0.318 **[0.424]
SECIS	76	1	**0.651**	0.658	0.645	0.520	0.257	0.224	0.329	0.191	0.480	**0.487**
7SK	184	1	**0.041**	0.043	0.038	0.024	0.916	0.060	0.011	0.802	0.188	**0.038**

Alignments not classified as structured RNA by RNAz												

5S rRNA	525	1	0.793	0.821	0.766	0.717	0.130	0.153	0.552	0.057	0.390	-
5.8S rRNA	1000	1	0.853	0.892	0.814	0.771	0.092	0.137	0.602	0.032	0.366	-
tRNA	1	1	1/1	1/1	1/1	1/1	0/1	0/1	1/1	0/1	0/1	-
miRNA	0	-	-	-	-	-	-	-	-	-	-	-
snoRNA (C/D)	4228	105	0.563 [0.397]	0.595 [0.399]	0.532 [0.414]	0.480 [0.420]	0.353 [0.363]	0.167 [0.236]	0.340 [0.394]	0.245 [0.316]	0.415 [0.364]	-
snoRNA (H/ACA)	1993	36	0.788 [0.251]	0.812 [0.244]	0.763 [0.291]	0.735 [0.314]	0.157 [0.203]	0.108 [0.233]	0.644 [0.370]	0.081 [0.169]	0.274 [0.339]	-
spliceos. RNA	2944	4	0.632 [0.287]	0.669 [0.287]	0.595 [0.289]	0.560 [0.314]	0.301 [0.261]	0.139 [0.071]	0.422 [0.338]	0.203 [0.200]	0.375 [0.180]	-
euk. SRP RNA	3	1	3/3	3/3	3/3	3/3	0/3	0/3	3/3	0/3	0/3	-
nucl. RNaseP	2	1	2/2	2/2	2/2	2/2	0/2	0/2	1/2	0/2	1/2	-
RNase MRP	0	-	-	-	-	-	-	-	-	-	-	-
IRES	265	13	0.506 [0.454]	0.521 [0.454]	0.491 [0.454]	0.468 [0.411]	0.457 [0.450]	0.075 [0.276]	0.436 [0.401]	0.353 [0.411]	0.211 [0.418]	-
SECIS	43	1	0.686	0.698	0.674	0.593	0.174	0.233	0.302	0.070	0.628	-
7SK	630	1	0.127	0.152	0.102	0.063	0.798	0.139	0.018	0.640	0.342	-

*N*_*a*_: number of alignments in test set, *N*_*c*_: number of different RNA classes, *A*: accuracy, which is defined as the fraction of correctly classified input alignments, *A*_+_: accuracy of alignments in reading direction of ncRNA, *A*_-_: accuracy of reverse complementary alignments, *u*: fraction of undecided alignments, 1 - *A *- *u*: fraction of misclassified alignments, *A*(RNAz): fraction of alignments correctly classified by taking the strand with the largest RNAz probability as the strand of the ncRNA. Standard deviations for RNA families with alignments from different classes are given in brackets. Note, that in case *c *= 0 no undecided alignments are observed.

To evaluate the performance of RNAstrand on alignments which have not been identified as structured RNA by RNAz, we constructed a second test set which only consists of alignments not classified as structured RNA by RNAz in both reading directions. This resulted in 207 families meeting the criteria described in the first paragraph of this section. The corresponding distributions are shown in the supplementary material (see Additional file 1). For those alignments a dramatic decrease of structure stability and conservation is observed which leads to smaller descriptor values (see Additional file 1). Hence, the classification performance is worse compared to RNAz-positive alignments (Table 1). However, for the majority of alignments the correct reading direction was inferred.

Performance measures depending on the number of sequences in the input alignment, the length as well as the mean pairwise identity of the sequences are given in Table 2. The number of sequences of an alignment does not influence prediction performance significantly. But the more the sequences are conserved the better the overall classification accuracy. The fraction of correctly classified alignments is also very high in case of long sequences. For alignments of 100 to 200 nt length the accuracy is biased to miRNAs, which are well classified by RNAstrand.

**Table 2:** Accuracies depending on different alignment features.

			*c *= 0	
alignment feature	*N*	*A*	*A*_+_	*A*_-_
*N*_*S *_= 2	4487	0.824	0.829	0.819
*N*_*S *_= 3	5311	0.833	0.830	0.837
*N*_*S *_= 4	6388	0.828	0.830	0.827
*N*_*S *_= 5	7234	0.797	0.805	0.789
*N*_*S *_= 6	7500	0.832	0.835	0.829

50 ≤ sequence identity < 70	13187	0.799	0.799	0.799
70 ≤ sequence identity < 80	12152	0.827	0.832	0.823
80 ≤ sequence identity < 90	5550	0.865	0.871	0.859
90 ≤ sequence identity < 100	31	0.903	0.871	0.935

40 ≤ length ≤ 100	11191	0.768	0.773	0.763
101 ≤ length ≤ 200	14180	0.853	0.856	0.851
201 ≤ length ≤ 300	1697	0.637	0.641	0.634
301 ≤ length ≤ 400	3852	0.945	0.945	0.945

all alignments	30920	0.822	0.825	0.819

Performance of RNAstrand depending on various alignment features, i.e. number of sequences (*N*_*S*_), sequence identity and alignment length. *N *: number of alignments in the test sets, *A*: accuracy, which is defined as the fraction of correctly classified input alignments, *A*_+_: accuracy of alignments in reading direction of ncRNA, *A*_-_: accuracy of reverse complementary alignments.

The results highlight that our classification task has an intrinsic symmetry: the fraction of correctly classified alignments for the "plus strand" of a ncRNA should be similar to the accuracy of the "minus strand". However, we observe a small but noticeable bias to predict that the ncRNA lies in same reading direction as the input alignment (Table 1). The SVM model was trained with different alignments in the positive and negative training sets, which results in an asymmetric model. If the same alignments, but in different directions, were taken for training, the SVM model would be exactly symmetric. But training data should be independent in the different classes, hence we refrained from enforcing this exact symmetry to avoid potential overtraining artifacts. Another possibility to avoid asymmetry would be to take the averaged SVM decision values of both reading directions as the final decision. But this has an unknown effect on the probability estimates.

**Figure 3:**
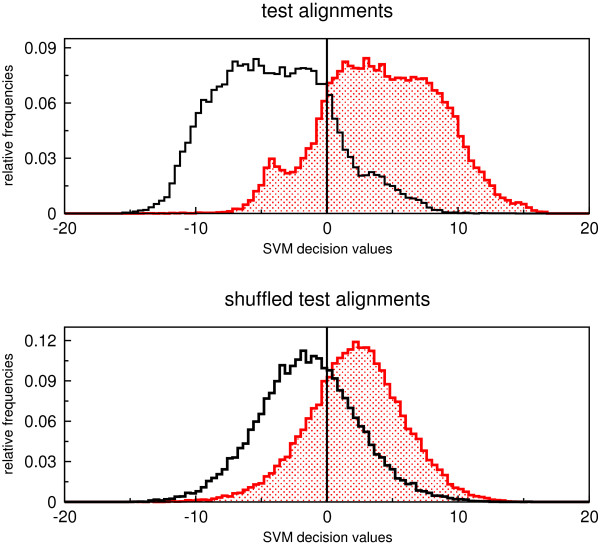
**Histogram of SVM decision values**. Distribution of SVM decision values of RNAz-positive alignments. The upper histogram belongs to all alignments of the test set. Whereas the lower one shows the distribution of the decision values for shuffled alignments. Columns of the test alignments were randomly permuted to create shuffled alignments. Red dotted bins denote alignments where the ncRNA has the same reading direction as the alignment. Black bins belong to alignments where the ncRNA is contained in the reverse complement. Note that the shuffling procedure does not completely destroy the direction information.

**Figure 4:**
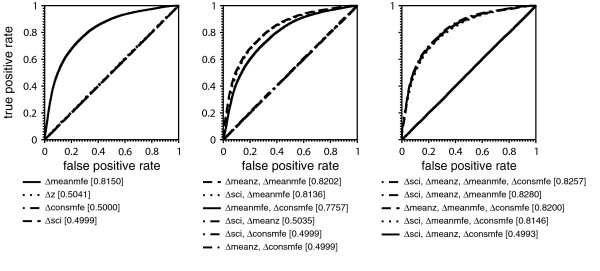
**Receiver operating characteristic of all descriptor combinations for shuffled alignments**. ROC curves of all descriptor combinations for shuffled alignments. Columns of test alignments were randomly permuted to create shuffled alignments. Corresponding *AUC *is given in brackets. ROC curves were computed by training a SVM model for each descriptor combination and testing the model on shuffled alignments by utilizing plotroc.py of the libsvm 2.8 package [18]. Training was done with the original training set for RNAstrand. SVM parameter and kernel did not change, i.e. a radial basis function kernel with parameters *C *= 128 and *γ *= 0.5 were used.

The distribution of decision values of the SVM is shown in Fig. 3. The majority of alignments were classified correctly. Most of them have large absolute decision values stating that they belong to the corresponding class with high probability. If RNAstrand is applied to shuffled alignments the decision values are more concentrated around 0, but most of them are still classified correctly. To explain this observation we checked which combination of descriptors performs best on shuffled alignments. We trained a SVM model for each possible descriptor combination and calculated the true and false positive rates at different decision levels by using plotroc.py of the libsvm 2.8 package [18]. The corresponding ROC curves are given in Fig. 4 and indicate that except of Δ*meanmfe *all descriptors classify shuffled alignments randomly. Individual shuffled sequences, presumably by virtue of their base composition (see Additional file 1), still contain information on the reading direction of the structured RNA which is captured by Δ*meanmfe*. This observation implies that RNAstrand must not be used for alignments that do not contain structured RNAs. In other words, RNAstrand cannot be used to infer an ncRNA on the grounds that it returned a preferred reading direction for a non-structured input alignment. We could have also removed Δ*meanmfe *from the set of descriptors, because of this bias. However, due to its high sensitivity (Fig. 1) it seems preferable to keep it as descriptor, in particular since RNAstrand is designed to operate on structured RNAs only. 

**Figure 5:**
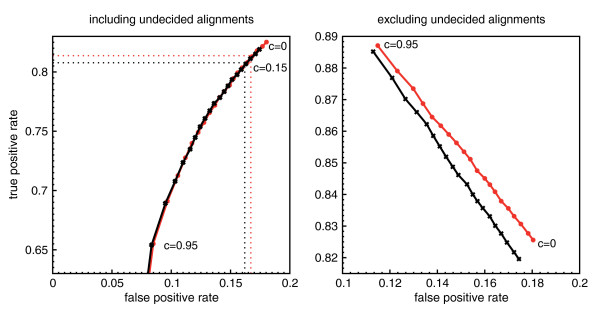
**Receiver operating characteristic of test alignments**. False positive rates of RNAz-positive test alignments versus true positive rates at different cutoff levels *c*. The left plot depicts rates in case undecided alignments are included in the calculation. Meaning that the true positive rate is defined as tptp+fn+u
 MathType@MTEF@5@5@+=feaafiart1ev1aaatCvAUfKttLearuWrP9MDH5MBPbIqV92AaeXatLxBI9gBaebbnrfifHhDYfgasaacH8akY=wiFfYdH8Gipec8Eeeu0xXdbba9frFj0=OqFfea0dXdd9vqai=hGuQ8kuc9pgc9s8qqaq=dirpe0xb9q8qiLsFr0=vr0=vr0dc8meaabaqaciaacaGaaeqabaqabeGadaaakeaadaWcaaqaaiabdsha0jabdchaWbqaaiabdsha0jabdchaWjabgUcaRiabdAgaMjabd6gaUjabgUcaRiabdwha1baaaaa@3861@, where *tp *denotes alignments which have been correctly classified to contain the ncRNA in the same reading direction as the input alignment. *fn *is the number of alignments which have been falsely classified to contain the ncRNA on the reverse complement, while *u *contains all alignments which contain the ncRNA in the same reading direction but RNAstrand were not able to predict a reading direction. False positive rate is defined respectively. The right handed plot discards unclassified alignments. Hence, the true positive rate is defined as tptp+fn
 MathType@MTEF@5@5@+=feaafiart1ev1aaatCvAUfKttLearuWrP9MDH5MBPbIqV92AaeXatLxBI9gBaebbnrfifHhDYfgasaacH8akY=wiFfYdH8Gipec8Eeeu0xXdbba9frFj0=OqFfea0dXdd9vqai=hGuQ8kuc9pgc9s8qqaq=dirpe0xb9q8qiLsFr0=vr0=vr0dc8meaabaqaciaacaGaaeqabaqabeGadaaakeaadaWcaaqaaiabdsha0jabdchaWbqaaiabdsha0jabdchaWjabgUcaRiabdAgaMjabd6gaUbaaaaa@360C@ and the false positive rate as fpfp+tn
 MathType@MTEF@5@5@+=feaafiart1ev1aaatCvAUfKttLearuWrP9MDH5MBPbIqV92AaeXatLxBI9gBaebbnrfifHhDYfgasaacH8akY=wiFfYdH8Gipec8Eeeu0xXdbba9frFj0=OqFfea0dXdd9vqai=hGuQ8kuc9pgc9s8qqaq=dirpe0xb9q8qiLsFr0=vr0=vr0dc8meaabaqaciaacaGaaeqabaqabeGadaaakeaadaWcaaqaaiabdAgaMjabdchaWbqaaiabdAgaMjabdchaWjabgUcaRiabdsha0jabd6gaUbaaaaa@35F0@. The curves for both SVM decision classes are given. Red curves denote alignments containing the ncRNA in the reading direction of the input alignment. Black curves belong to alignments which contain the ncRNA on the reverse complementary strand. The values of *c *range from 0 to 0.95 in steps of 0.05.

The best cutoff *c *can be found by plotting false positive rates versus true positive rates at different *c *(Fig. 5). If Youden's index *Y*, i.e., true positive rate minus false positive rate, is maximal, then the classification accuracy cannot be further improved by taking a larger cutoff [19]. We observe *Y*_max _≈ 0.644 for *c *≤ 0.15. Hence, a further increase of *c *leads to a worse proportion of correctly and falsely classified alignments. However, a large value of *c *assures that the predicted reading direction is with high probability the correct reading direction, see Table 1 and the r.h.s. of Fig. 5.

### 2.2 Comparison to naïve approaches

A naïve way to determine the likely reading direction is to score an alignment and its reverse complement using RNAz, EvoFold, or another tool for recognizing structured RNAs. This approach was taken e.g. in [1,2,4,5]. A manual inspection of the data, however, showed that this approach is problematic in particular in those cases where RNAz scores are high for both reading directions. This is the case in particular for microRNA precursors, but also for many other small house-keeping ncRNAs.

Table 1 gives the accuracy of RNAstrand compared to this simple approach, i.e., taking the strand with the larger RNAz probability. RNAstrand yields for all ncRNA types an improvement. The largest increase of classification accuracy is observed for miRNAs, RNase MRP, tRNAs, nuclear RNaseP and IRES. Table 3 shows that the reading direction is classified correctly in the majority of test alignments by RNAstrand. The misclassification rate of the naïve approach is two times higher than that of RNAstrand. 

Finally, we compared the prediction accuracy of RNAstrand with the strand prediction of EvoFold. Applying EvoFold to automatically created RNA alignments extracted from Rfam families is not easily feasible since EvoFold requires a meaningful phylogenetic tree (ideally estimated from neutrally evolving sites) as input. Such data are not available and cannot be generated easily for most combinations of Rfam sequences. The heuristic suggested in [2], namely to rescale a neighbor-joining tree generated from the input alignment, produced very poor classification results in most cases.

**Table 3:** Comparison of classification accuracies versus RNAz.

			Naïve RNAz-based classification	
			correct	incorrect
RNAstrand	fwd	correct	17961	7579
		incorrect	1570	3810

	rev	correct	17855	7521
		incorrect	1676	3868

	all	correct	35816	15100
		incorrect	3246	7678

Strand prediction of RNAstrand compared to naïve prediction of RNAz. The first row of the table refers to alignments of known ncRNA loci given in the direction of the ncRNA. The second row belongs to the corresponding reverse complementary alignments. The last row summarizes the first and second row.

Hence, we use instead the subset of known ncRNAs among the 48479 EvoFold predictions in human assembly hg17 [2].

A blast search with *E *< 1*e *- 10 against NonCode [20], Rfam [21], mirBase [22] and snoRNA-LBME-db [23] identified only 248 unique known ncRNA loci in human. (Note, that tRNAs and most snRNAs are multi-copy genes and hence were deliberately excluded from the data in [2]). To compare strand predictions of EvoFold with RNAstrand the multiz8way alignments of 202 loci, which are completely covered by a blast hit, were reconstructed. The majority (177) were identified to be miRNA precursors as most of the EvoFold predictions in ref. [2] are short conserved hairpins. The direction of the blast hit indirectly determines the strand of the known ncRNA when it is compared to the strand prediction of EvoFold. For 14 (13 miRNAs and 1 U6atac) loci the multiple alignments could not be reconstructed. The remaining 188 alignments were realigned and all which did not meet the prerequisites of RNAstrand were discarded: 15 alignments were shorter than the minimum length for which RNAstrand was trained with, 5 alignments had a mean pairwise identity smaller than 50%, and one alignment contained of too many gaps. This leaves 167 alignments for which the strand prediction of RNAstrand is compared to the strand prediction of EvoFold. Alignments containing more than 6 sequences were reduced to 6 sequences by rnazWindow.pl which optimizes the final alignment for a mean pairwise identity.

The numbers in Table 4 show that the strand prediction of EvoFold is comparable to the strand prediction of RNAstrand on this relative small test set, which is, however, dominated by microRNAs. We remark that EvoFold and RNAz are sensitive for ncRNAs of different base compositions and sequence similarities [3,24], so that neither of these programs can be (ab)used as universal strand-strand classificators.

**Table 4:** Comparison of classification accuracies versus EvoFold.

			Naïve EvoFold-based classification	
			correct	incorrect
RNAstrand	fwd	correct	123 [111;12]	16 [15;1]
		incorrect	17 [17; 0]	11 [8;3]

	rev	correct	121 [109;12]	12 [11;1]
		incorrect	19 [19; 0]	15 [12;3]

	all	correct	244 [220;24]	28 [26;2]
		incorrect	36 [36; 0]	26 [20;6]

Strand prediction of RNAstrand compared to naïve prediction of EvoFold. The first row of the table refers to alignments of known ncRNA loci given in the direction of the ncRNA. The second row belongs to the corresponding reverse complementary alignments. The last row summarizes the first and second row. First numbers in brackets give classifications of alignments containing miRNAs and second numbers belong to alignments containing other ncRNAs.

## 3 Discussion

RNA molecules and their reverse complements in general form fairly similar secondary structures [25]. For individual sequences, small differences between plus and minus strand arise from small asymmetries in the energy model [9]. In a multiple sequence alignment, GU pairs in an evolutionary conserved stem provide information on the correct reading direction since their reverse complement, AC, is not a canonical base pair. Nevertheless, it is a surprisingly hard problem to recognize the correct reading direction of a structured RNA from a multiple sequence alignment in practise. This is an important task in genome annotation, however, since without reliable strand information it is not even possible to determine whether an evolutionarily conserved secondary structure is located in an UTR or intron, or in an antisense transcript. The reading direction is also of obvious importance in context of recognizing class membership by means of short sequence motifs such as SMN-binding sites [26] or a Cajal body localization signal [27].

The RNAstrand tool presented in this contribution uses a SVM to predict strand information from a set of four thermodynamic features that can readily be computed for any multiple sequence alignment based on well-established energy parameters and dynamic programming algorithms. We show here that, together with basic information on the size, sequence and *GU *base pair variation in the input alignment, these features are sufficient to determine the reading direction of an RNA motif with an evolutionary conserved secondary structure. The tool RNAstrand achieves classification accuracies of 90% and above for most ncRNA families. On microRNAs, its performance is comparable to that of EvoFold. In applications to data from organisms for which not much genomic DNA has been sequenced, RNAstrand has the advantage that it does not require fairly accurate estimates of evolutionary distances as input.

The main area of application for a tool like RNAstrand is of course in large scale surveys for evolutionary conserved ncRNAs. RNAstrand achieves a 2-fold reduction of misclassifications on known ncRNAs compared to the naïve approach of determining the likely reading direction by comparing the scores of ncRNA detectors in both directions in the case of RNAz. It has therefore been integrated into the current release 1.0 of the RNAz package [28].

## Availability and requirements

Project name: RNAstrand

Project homepage: 

Operating system(s): platform independent

Programming language: C

Requirements: Vienna RNA Package  and the LIBSVM library for support vector machines 

License: GNU GPL.

Restrictions to use by non-academics: Note that a license is needed to *include *source code from the Vienna RNA Package in *commercial *software projects.

## Supplementary Material

Additional File 1 Supplementary material. Supplementary material to RNAstrand: reading direction of structured RNAs in multiple sequence alignments. Click here for file
