# Supplementary material for: RNAstrand: reading direction of structured RNAs in multiple sequence alignments
Source: Algorithms Mol Biol. 2007 May 31;2:6. doi: 10.1186/1748-7188-2-6 (PMC1892782; doi:10.1186/1748-7188-2-6)
Supplement: Additional File 1 — Supplementary material. Supplementary material to RNAstrand: reading direction of structured RNAs in multiple sequence alignments. [file 1748-7188-2-6-S1.pdf]

Supplementary Material
RNAstrand: reading direction of structured RNAs in multiple sequence alignments

Kristin Reiche*[1], Peter F. Stadler[1,2,3]

[1]Bioinformatics Group, Dept. of Computer Science, and Interdisciplinary Center for Bioinformatics, University of Leipzig, Härtelstraße 16-18, D-04107 Leipzig, Germany
[2]Institute for Theoretical Chemistry, University of Vienna, Währingerstraße 17, A-1090 Wien, Austria
[3]Santa Fe Institute, 1399 Hyde Park Rd., Santa Fe, NM 87501,USA

Email: Kristin Reiche - kristin@bioinf.uni-leipzig.de; Peter F. Stadler - studla@bioinf.uni-leipzig.de;

*Corresponding author

Distributions of sequence identity and number of sequences for tRNA and U70 snoRNA alignments:

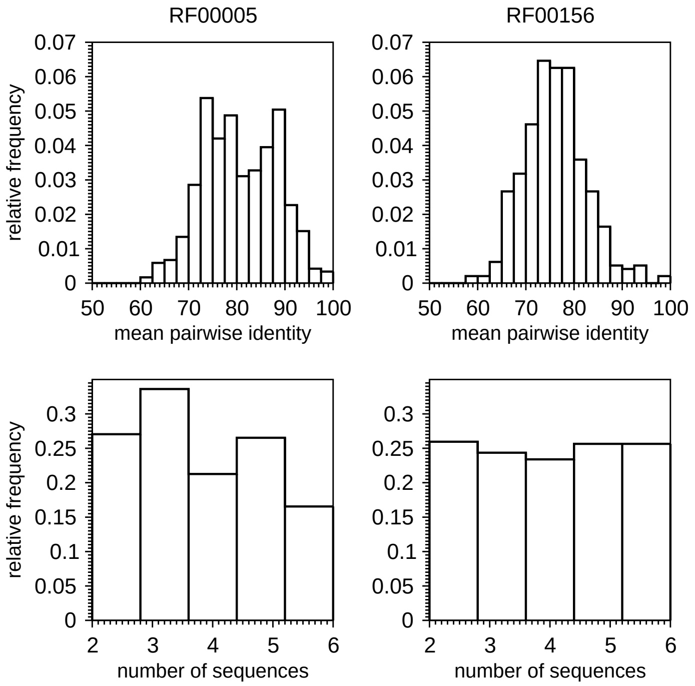

Figure 1: Histograms of mean pairwise sequence identity and number of sequences of tRNA and U70 snoRNAs alignments. Distributions do not differ significantly, hence smaller descriptor values of tRNA alignments are not explainable by different evolutionary distance between sequences nor by the number of sequences given in the alignment. They are completely explained by the different distributions of GU base pairs.

Features of training and test alignments:

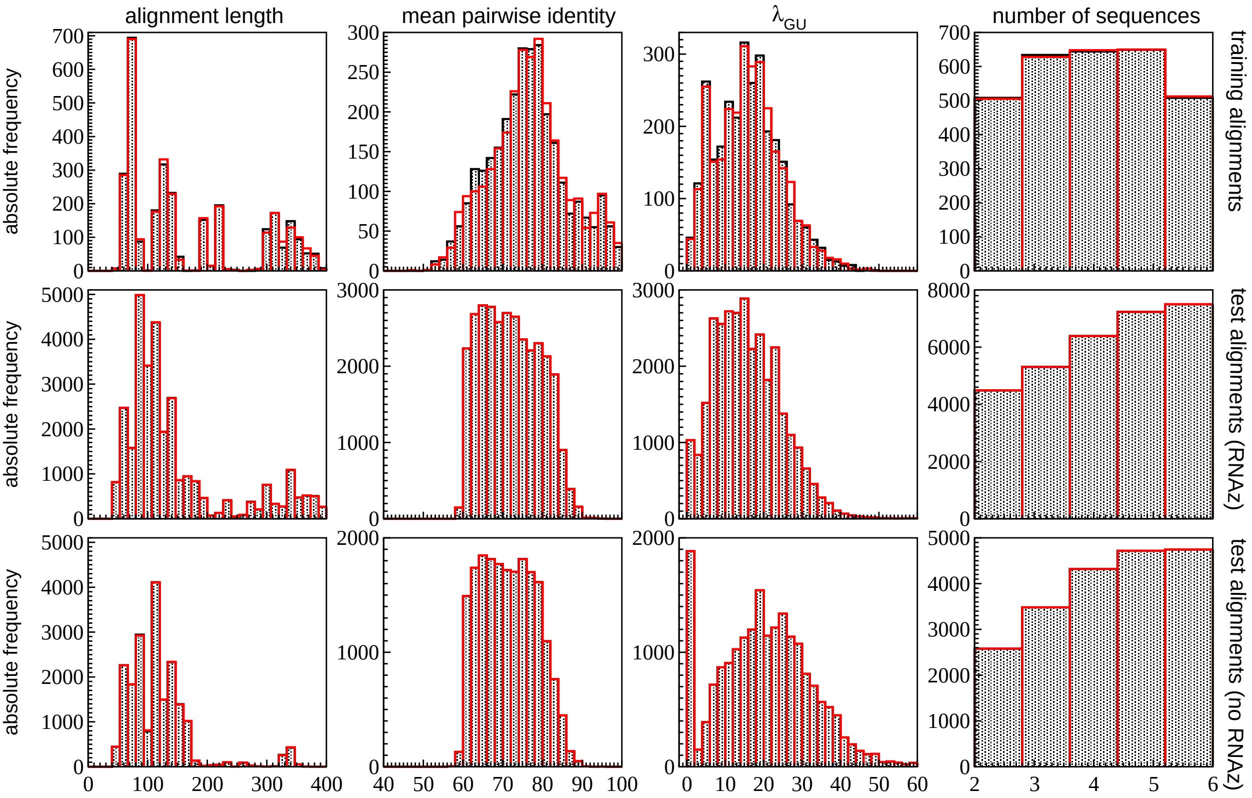

Figure 2: Distributions of length, mean pairwise identity, GU base pair content and number of sequences of the training alignments (first row), test alignments classified as structured RNA by RNAz (second row) and test alignments not classified as structured RNA by RNAz (third row). Red bins belong to alignments having the ncRNA in the reading direction of the alignment. Black bins belong to their realigned reverse complements.

Distribution of RNAstrand score D:

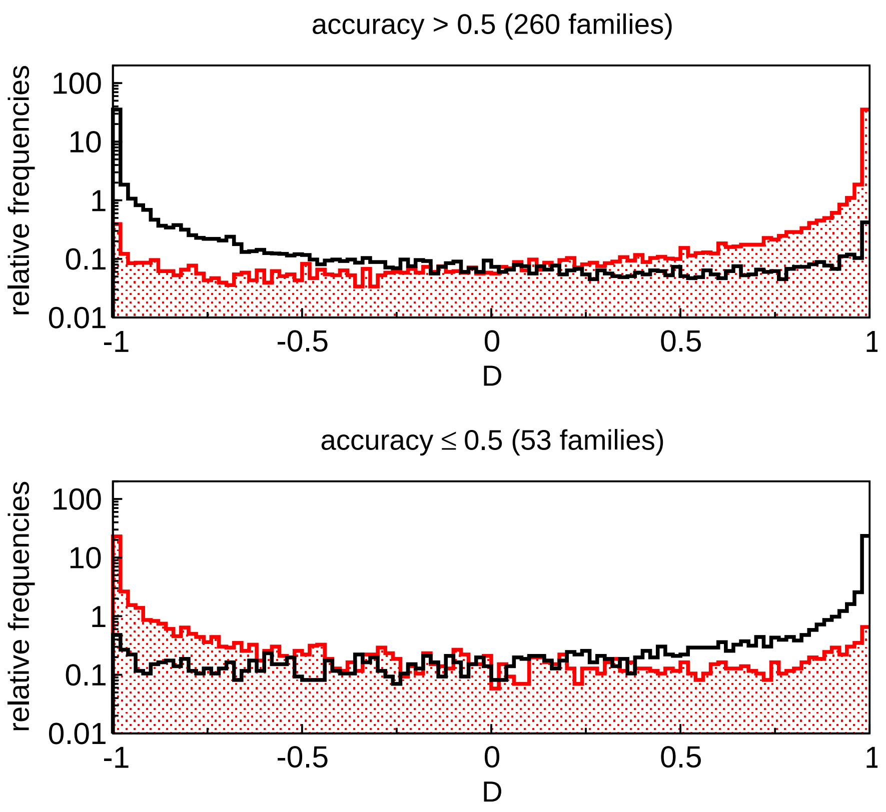

Figure 3: Distribution of RNAstrand score D for RNAz-positive alignments. The upper picture shows the distribution of D of families with an accuracy of at least 50%, while the lower picture depicts the distribution of D of families with an accuracy less or equal than 50%. Red dotted bins denote alignments containing the ncRNA in the reading direction of the alignment, while black bins correspond to alignments having the ncRNA in the reverse complement. The distribution of D of alignments which hold the ncRNA in the same reading direction is nearly symmetric to the distribution of D of alignments containing the ncRNA in the reverse complement.

Structure stability and conservation of 7SK RNA:

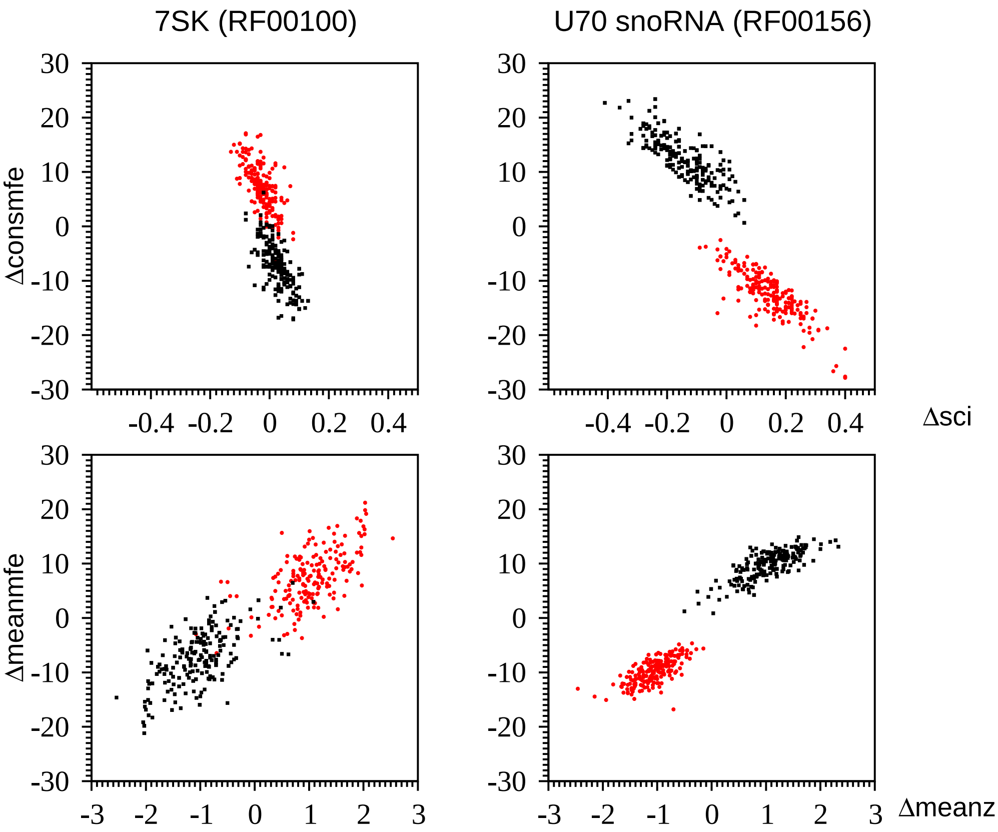

Figure 4: Correlation of $\triangle$*consmfe* versus $\triangle$*sci* and $\triangle$*meanmfe* versus $\triangle$*meanz*. The difference in stability of individual and consensus sequences demonstrates that the predicted minimum free energy structures of the reverse complementary sequences of 7SK are more stable than 7SK itself. Red dots correspond to alignments having ncRNA in the reading direction of the alignment and black dots are alignments containing the ncRNA in their reverse complement.

Distribution of descriptor values for test alignments:

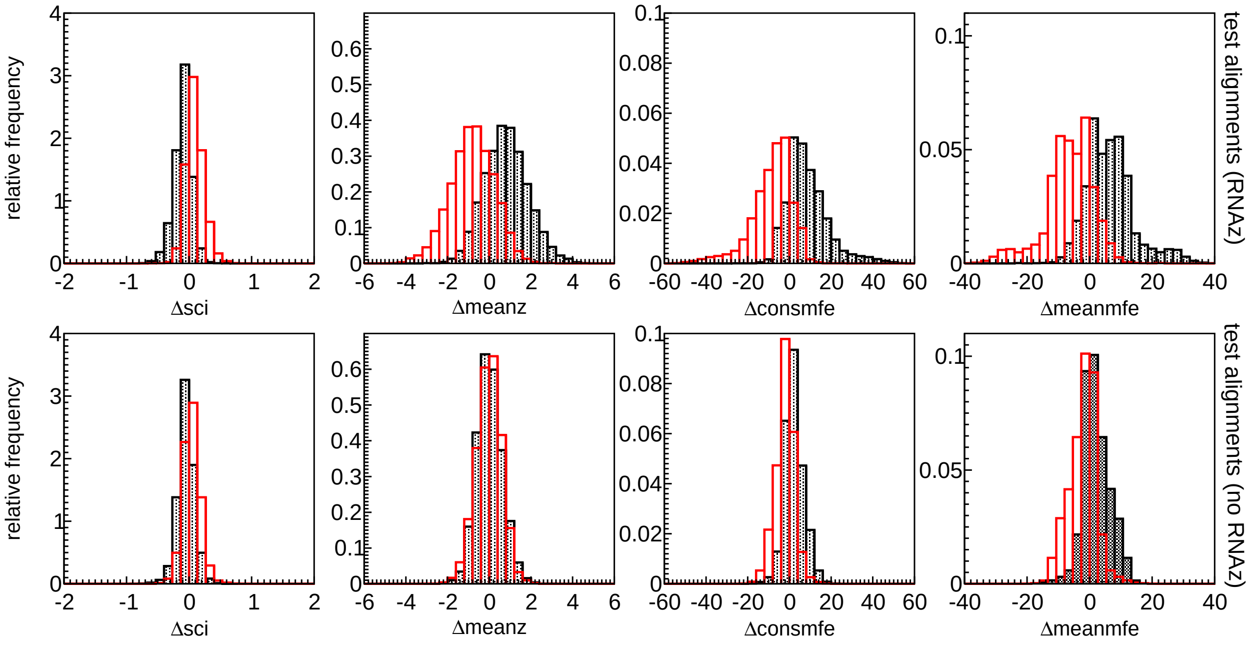

Figure 5: Distribution of descriptor values of test alignments which have been classified as structured RNA by `RNAz` (first row) versus alignments which were not classified as structured RNA (second row). Again, red bins denote alignments containing the ncRNA in the reading direction of the alignment, while black bins correspond to alignments having the ncRNA in the reverse complement.

GU base pair fraction distribution of original and shuffled alignments:

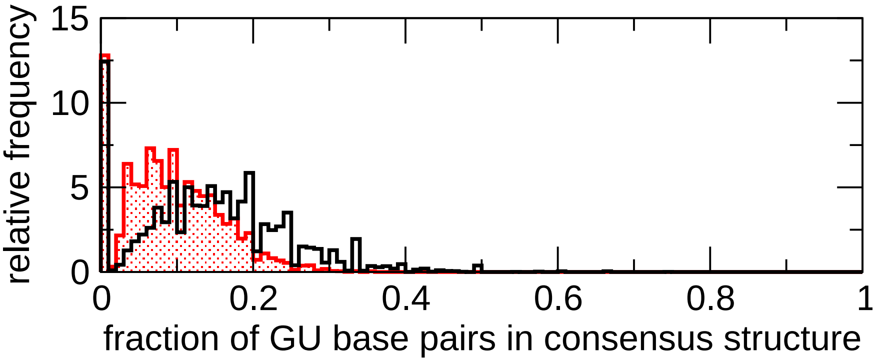

Figure 6: Fraction of GU base pairs of consensus secondary structure of alignments in reading direction of ncRNA. Red bins correspond to original test alignments in the reading direction of the ncRNA, while black bins belong to their shuffled alignments. Fraction is defined as ratio of GU base pairs and all base pairs in consensus. The GU base pair fraction of shuffled alignments is shifted to higher values compared to the original alignments. This may be a possible explanation why shuffled alignments are still classified correctly, as GU pairs have a large effect on identifying reading direction. Note that the fraction of GU base pairs is not normalized by the length of the alignment, but by the number of all base pairs in consensus structure.

RNA families discarded from test set:

Reason	N	RFAM accession numbers
No direction identified as ncRNA by RNAz	113	RF00016 RF00033 RF00038 RF00044 RF00046 RF00049 RF00060 RF00065 RF00071 RF00081 RF00085 RF00087 RF00088 RF00089 RF00098 RF00105 RF00109 RF00115 RF00120 RF00136 RF00141 RF00145 RF00146 RF00151 RF00152 RF00153 RF00157 RF00158 RF00172 RF00179 RF00180 RF00189 RF00191 RF00197 RF00200 RF00202 RF00205 RF00222 RF00225 RF00227 RF00228 RF00232 RF00270 RF00271 RF00273 RF00274 RF00278 RF00280 RF00281 RF00282 RF00285 RF00287 RF00289 RF00292 RF00293 RF00294 RF00295 RF00301 RF00303 RF00307 RF00309 RF00311 RF00312 RF00315 RF00316 RF00322 RF00324 RF00325 RF00331 RF00335 RF00339 RF00342 RF00343 RF00348 RF00350 RF00351 RF00352 RF00355 RF00356 RF00382 RF00383 RF00389 RF00390 RF00395 RF00399 RF00419 RF00439 RF00440 RF00441 RF00448 RF00449 RF00454 RF00459 RF00460 RF00461 RF00462 RF00463 RF00471 RF00473 RF00474 RF00475 RF00476 RF00477 RF00479 RF00483 RF00487 RF00488 RF00494 RF00495 RF00496 RF00501 RF00503 RF00509
No alignments between 40nt and 400nt	8	RF00024 RF00028 RF00032 RF00037 RF00177 RF00210 RF00453 RF00469
No alignments with mean pairwise identity above 60%	2	RF00022 RF00130
Family consist of only one or two sequences	67	RF00058 RF00083 RF00116 RF00117 RF00118 RF00119 RF00122 RF00125 RF00155 RF00184 RF00186 RF00187 RF00188 RF00192 RF00193 RF00194 RF00196 RF00201 RF00207 RF00208 RF00217 RF00223 RF00224 RF00261 RF00268 RF00290 RF00291 RF00297 RF00299 RF00300 RF00302 RF00305 RF00306 RF00310 RF00313 RF00314 RF00317 RF00318 RF00321 RF00323 RF00326 RF00327 RF00336 RF00338 RF00346 RF00358 RF00365 RF00367 RF00372 RF00385 RF00397 RF00400 RF00407 RF00417 RF00426 RF00433 RF00437 RF00443 RF00457 RF00466 RF00481 RF00484 RF00491 RF00493 RF00498 RF00500 RF00502

Table 1: A list of all RFAM families which were discarded from the test set for RNAstrand.
